# Supplementary material for: Regulation of a PRMT5/NF-κB Axis by Phosphorylation of PRMT5 at Serine 15 in Colorectal Cancer
Source: Int J Mol Sci. 2020 May 23;21(10):3684. doi: 10.3390/ijms21103684 (PMC7279388; doi:10.3390/ijms21103684)
Supplement: Supplementary file 1 [file ijms-21-03684-s001.pdf]

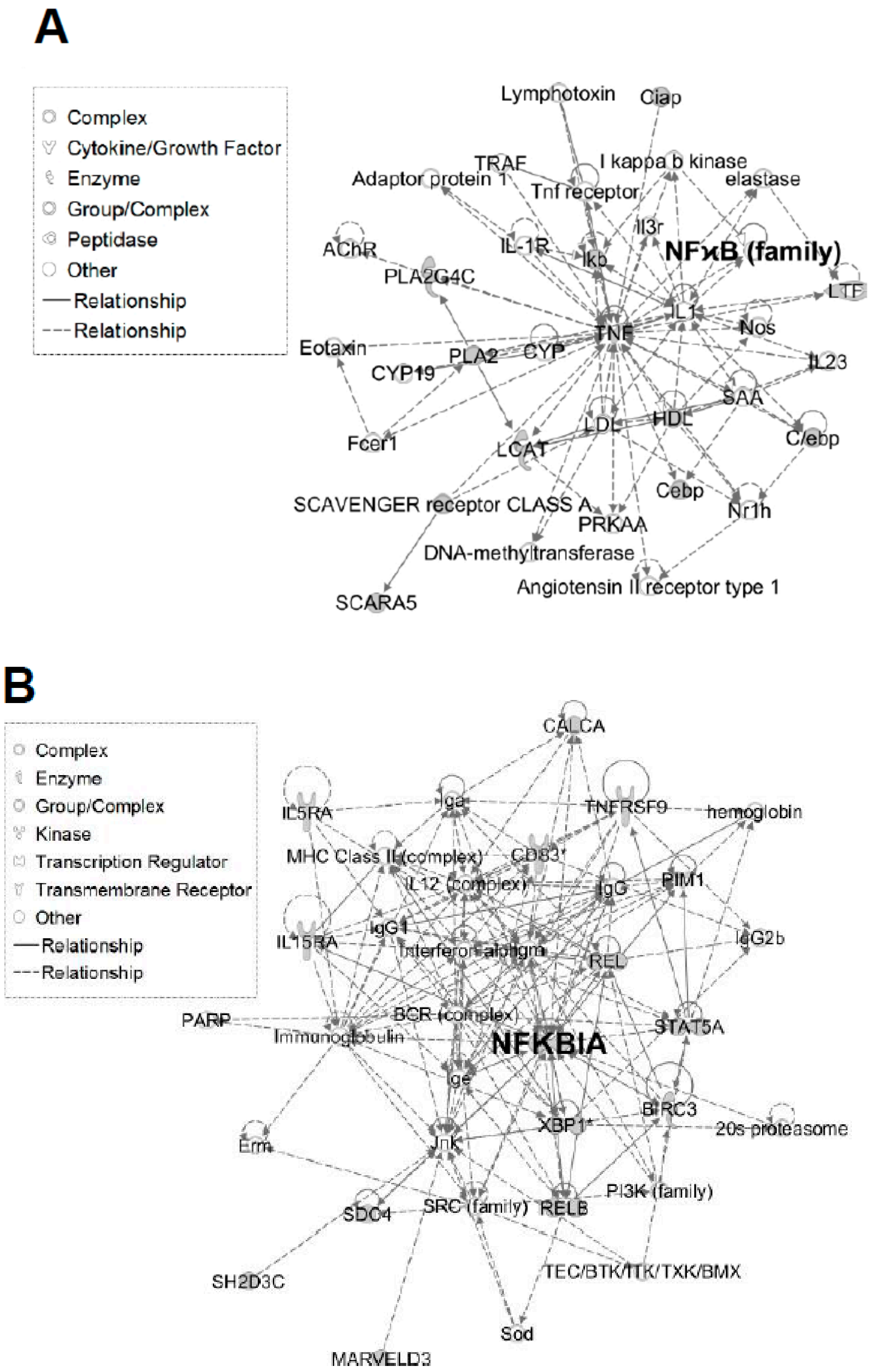


Figure S1. Ingenuity Pathway Analysis (IPA). (A) and (B) IPA representative networks showing genes regulated by S15A with NF-κB as one of the critical nodes in these networks.

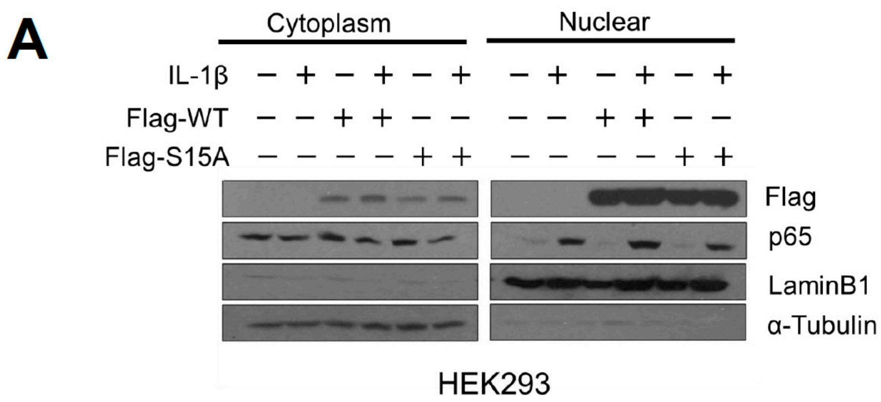


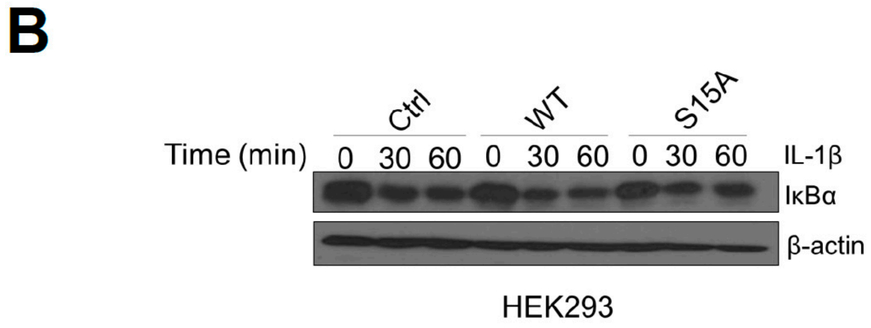


Figure S2. Effect of S15A mutant on PRMT5 subcellular localization, p65 nuclear translocation and IκB$\alpha$ degradation. (A) Cell fractionation assay, showing subcellular compartmentalization of Flag-PRMT5 and Flag-S15A in HEK293 cells. Western blot was probed with anti-FLAG, p65, LaminB1 and $\alpha$-tubulin antibodies. (B) Western blot, showing IL-1β-induced IκB$\alpha$ degradation pattern in WTPRMT5 and S15A HEK293 cells.

| ACCESSION NO. | TargetID | S15A-PRMT5 +IL-1β/WT+IL-1β |
|---|---|---|
| NM_001115.1 | ADCY8 | 0.45 |
| NM_014391.2 | ANKRD1 | 0.38 |
| NM_020373.2 | ANO2 | 0.15 |
| XR_041624.1 | C10ORF85 | 0.36 |
| XR_041485.1 | C13ORF29 | 0.27 |
| XM_001726191.1 | C19ORF29OS | 0.00 |
| NM_178342.2 | C3ORF35 | 0.50 |
| NM_018452.3 | C6ORF35 | 0.32 |
| NM_145028.3 | C6ORF81 | 0.16 |
| NM_152786.1 | C9ORF43 | 0.33 |
| NM_001742.2 | CALCR | 0.24 |
| NM_175931.1 | CBFA2T3 | 0.28 |
| NM_138414.1 | CCDC101 | 0.41 |
| NM_015603.1 | CCDC9 | 0.48 |
| NM_004591.1 | CCL20 | 0.37 |
| NM_207007.2 | CCL4L2 | 0.47 |
| NM_145057.2 | CDC42EP5 | 0.41 |
| NM_000735.2 | CGA | 0.33 |
| NM_000737.2 | CGB | 0.45 |
| NM_024111.2 | CHAC1 | 0.00 |
| NM_015557.1 | CHD5 | 0.27 |
| NM_024944.2 | CHODL | 0.47 |
| NM_138429.1 | CLDN15 | 0.49 |
| NM_080645.2 | COL12A1 | 0.22 |
| NM_002089.3 | CXCL2 | 0.29 |
| NM_016229.3 | CYB5R2 | 0.36 |
| NM_000779.2 | CYP4B1 | 0.31 |
| NR_024064.1 | DAD1L | 0.39 |
| NM_006557.4 | DMRT2 | 0.28 |
| NR_024595.1 | DNM1P35 | 0.06 |
| NM_133637.1 | DQX1 | 0.37 |
| NM_004428.2 | EFNA1 | 0.48 |
| NM_007036.2 | ESM1 | 0.22 |
| NM_153606.2 | FAM71A | 0.50 |
| NM_001012426.1 | FOXP4 | 0.06 |
| NM_000148.2 | FUT1 | 0.35 |

| | | |
|---|---|---|
| NM_002068.1 | GNA15 | 0.40 |
| XM_935238.1 | GOLGA8F | 0.45 |
| BX109627 | HS.130639 | 0.46 |
| AI801879 | HS.144030 | 0.14 |
| BU633914 | HS.25555 | 0.27 |
| AK026734 | HS.287720 | 0.34 |
| AF339771 | HS.344872 | 0.19 |
| CD695721 | HS.538157 | 0.00 |
| AI253067 | HS.541845 | 0.20 |
| U10515 | HS.544238 | 0.46 |
| AI628074 | HS.545238 | 0.13 |
| NM_130770.2 | HTR3C | 0.28 |
| NM_172200.1 | IL15RA | 0.17 |
| NM_001012633.1 | IL32 | 0.49 |
| NM_000564.2 | IL5RA | 0.50 |
| NM_000584.2 | IL8 | 0.48 |
| NM_002195.1 | INSL4 | 0.30 |
| XM_934728.1 | KIAA0565 | 0.09 |
| NM_138343.2 | KLC4 | 0.02 |
| NM_002774.3 | KLK6 | 0.34 |
| NM_004139.2 | LBP | 0.29 |
| NM_001010939.1 | LIPJ | 0.18 |
| NM_033029.2 | LMLN | 0.15 |
| XM_001724965.1 | LOC100129268 | 0.13 |
| XM_001716704.1 | LOC100130288 | 0.15 |
| XM_001718675.1 | LOC100130705 | 0.50 |
| XM_001714361.1 | LOC100131999 | 0.18 |
| XR_038987.1 | LOC100132496 | 0.37 |
| XM_001724630.1 | LOC100132716 | 0.05 |
| XM_001726146.1 | LOC100132839 | 0.06 |
| XM_001721522.1 | LOC100134009 | 0.18 |
| XM_001720931.1 | LOC100134041 | 0.14 |
| XM_001715304.1 | LOC100134081 | 0.00 |
| XM_001721704.1 | LOC100134170 | 0.19 |
| XM_001714134.1 | LOC100134499 | 0.00 |
| NM_178514.3 | LOC283487 | 0.26 |
| XM_944838.2 | LOC285733 | 0.27 |
| XM_939888.1 | LOC339742 | 0.49 |
| XM_370865.4 | LOC388122 | 0.23 |

| | | |
|---|---|---|
| XM_374766.2 | LOC399715 | 0.11 |
| XM_495854.3 | LOC440013 | 0.10 |
| XM_001717499.1 | LOC642076 | 0.04 |
| NR_024495.1 | LOC642826 | 0.40 |
| XM_927139.1 | LOC643869 | 0.14 |
| XM_933938.2 | LOC643872 | 0.38 |
| XM_928663.1 | LOC645649 | 0.03 |
| XR_037491.1 | LOC646808 | 0.46 |
| XM_943707.1 | LOC649431 | 0.33 |
| XM_941853.1 | LOC652416 | 0.00 |
| XM_928640.1 | LOC653651 | 0.39 |
| XM_001126803.1 | LOC728185 | 0.36 |
| XR_015405.1 | LOC728895 | 0.43 |
| XM_001130993.1 | LOC729675 | 0.23 |
| XM_001714434.1 | LOC730376 | 0.40 |
| NM_002343.2 | LTF | 0.01 |
| NM_012323.2 | MAFF | 0.44 |
| NM_005204.2 | MAP3K8 | 0.39 |
| NM_052858.3 | MARVELD3 | 0.33 |
| NM_033290.1 | MID1 | 0.33 |
| NR_030209.1 | MIR518E | 0.31 |
| NM_173855.3 | MORN3 | 0.40 |
| NM_013404.3 | MSLN | 0.21 |
| NM_003828.2 | MTMR1 | 0.45 |
| NM_001005474.1 | NFKBIZ | 0.47 |
| NM_001080379.1 | PACRG | 0.01 |
| NM_000438.3 | PAX3 | 0.27 |
| NM_003706.1 | PLA2G4C | 0.41 |
| NM_002658.2 | PLAU | 0.44 |
| XM_940486.1 | PLEKHA2 | 0.26 |
| NM_014330.2 | PPP1R15A | 0.46 |
| NM_022114.2 | PRDM16 | 0.11 |
| NM_000963.1 | PTGS2 | 0.32 |
| NM_000963.1 | PTGS2 | 0.40 |
| NM_001024455.2 | RGAG4 | 0.43 |
| NM_184237.1 | RNPC2 | 0.48 |
| NM_001007098.1 | SCP2 | 0.40 |
| NM_000450.1 | SELE | 0.22 |
| NM_013386.3 | SLC25A24 | 0.41 |

| | | |
|---|---|---|
| NR_003237.1 | SNORD113-9 | 0.34 |
| XM_291729.7 | TAF3 | 0.38 |
| NM_031898.1 | TEKT3 | 0.16 |
| NM_001001524.2 | TM6SF2 | 0.10 |
| NM_001097620.1 | TMEM184A | 0.08 |
| NM_002160.2 | TNC | 0.45 |
| NM_002160.1 | TNC | 0.44 |
| NM_006290.2 | TNFAIP3 | 0.28 |
| NM_001561.4 | TNFRSF9 | 0.50 |
| NM_033229.2 | TRIM15 | 0.41 |
| NM_020810.2 | TRMT5 | 0.21 |
| NM_001080419.1 | UNK | 0.50 |
| NM_030570.2 | UPK3B | 0.39 |
| NM_001078.2 | VCAM1 | 0.15 |
| NM_206923.1 | YY2 | 0.30 |
| NM_145271.3 | ZNF688 | 0.18 |

Figure S3. Full list of genes downregulated by the S15A-PRMT5 mutation. Fold change is represented as S15A-PRMT5 +IL-1β/WT+IL-1β $\leq 0.5$
